# Correlation between OCT-angiography and photopic negative response in patients with primary open angle glaucoma

**DOI:** 10.1007/s10792-022-02588-9

**Published:** 2022-11-28

**Authors:** Ahmed Abdelshafy Tabl, Marwa Abdelshafy Tabl

**Affiliations:** Department of Ophthalmology, Faculty of Medicine, Benha University, Farid Nada St., Benha, 13512 Egypt

**Keywords:** Photopic negative response, Open angel glaucoma, OCT-angiography, Retinal nerve fiber layer

## Abstract

**Purpose:**

To evaluate the association between OCT-angiography (OCTA) and photopic negative response (PhNR) in open angle glaucoma (OAG) patients and assess the diagnostic accuracy of these parameters in early detection of glaucoma.

**Methods:**

A total of 152 eyes were enrolled in this study, 28 eyes with mild POAG (group I), 44 eyes with moderate-severe POAG (group 2) & 80 eyes of healthy subjects (control group). Full ophthalmological examination, OCTA and PhNR measurements were underwent for all participants. RNFL, GCC thicknesses, PhNR (implicit time and amplitude) were recorded. The superficial and deep capillary plexus vessel density (SCP-VD%, DCP-VD%) were measured by using 6 × 6 mm macula OCTA scans. The peripapillary vessel density (RPC-VD %) were measured by using 4.5 × 4.5 mm optic disk head OCTA scans.

**Results:**

There were reduction of the median Interquartile range (IQR) thickness of the GCC and RNFL in OAG eyes versus normal (P < 0.001). RPC-VD%, SCP-VD % and DCP-VD% were significantly reduced in OAG eyes versus normal (P < 0.001). Increased OAG severity was associated with more reduction in PhNR amplitude and increased implicit time. Reduced PhNR amplitude and prolonged latency were significantly correlated with reduced vascular parameters. The RCP-VD and PhNR amplitude demonstrated higher diagnostic accuracy (98.7) with the largest AUC and higher sensitivity and specificity (100% & 98.7%, respectively), followed by the PhNR implicit time with (AUC = 0.995) with a diagnostic accuracy 98.7. The SCP-VD, RNFL and GCC thickness had a diagnostic accuracy of (75.0, 81.6 & 84.2), respectively (P < 0.001).

**Conclusions:**

OCTA vascular parameters displayed significant positive correlation with PhNR amplitude and significant negative correlation with PhNR implicit time. OCTA and PhNR parameters showed a high diagnostic accuracy for detection of glaucoma, and both may provide promising insight in early detection of glaucoma.

This study was retrospectively registered on ClinicalTrials.gov (identifier, NCT05104294).

## Introduction

Open angle glaucoma (OAG) is considered the second leading cause of irreversible vision loss globally [1]. It is an optic neuropathy associated with progressive degeneration and loss of the retinal ganglion cell (RGC) and the retinal nerve fiber layer (RNFL), with interrelated neuroretinal rim excavation and visual field defects [1, 2].

The pathophysiology and factors contributing to glaucoma progression have not been fully understood [3]. Several recent studies suggested that vascular dysfunction plays a significant role on glaucoma pathogenic concept. Decreased ocular blood flow may be associated and even predict glaucomatous nerve damage [4, 5].

Optical Coherence Tomography Angiography (OCTA) and adaptive optics-OCT are promising current tools for diagnosing and management of glaucomatous patients. OCTA can measure the blood flow within the retina and the optic nerve, and thus can assess the degree of glaucomatous damage and evaluate the health of the ganglion cells and their axons [6–8].

Recent publications using OCTA had mainly focused on evaluation of the microcirculation inside the optic disk and in the peripapillary area and displayed reduction of the peripapillary microcirculation [9–11]. Recent glaucoma studies have also paid attention to the macula as it has the highest density of the RGC. Vascular and functional measures of the inner macula may be a useful tool in assessment of glaucoma progression [12–15].

The photopic negative response (PhNR) is an objective, minimally-invasive test that reflect the function of RGC and their axons. It can be reduced in glaucoma and disorders that affect the inner-most retina [16, 17].

Structural OCT measurements have limited diagnostic ability in identifying early stages of OAG, especially in patients with atypical optic nerves as in high myopia or uveitis and have moderate correlation with visual field testing [11, 18–20].

Controversy regarding the associations between the vascular, functional and structural parameters in OAG is still present and the sensitivity of these parameters varies greatly. Some studies have found stronger associations between vascular and functional parameters rather than structural ones [6, 7, 20, 21] and others found a stronger association between the vascular and structural parameters [18, 22–24]. Further studies are still needed to elucidate these correlations.

The aim of the present study was to evaluate the correlations between vascular, functional and structural changes of the peripapillary RNFL and macular/ganglion cell complex in OAG patients and assess the diagnostic accuracy of OCTA and PhNR parameters in early detection of glaucoma changes.

## Materials and methods

### Patients

This cross-sectional comparative study included one hundred fifty-two eyes of 76 participants. All participants were recruited from the outpatient clinics of Benha University Hospital. 72 eyes of 36 patients with primary open angle glaucoma (glaucoma group) with a mean age of (40.2 ± 4.39) and 80 eyes of 40 healthy subjects (control group) with a mean age of (40.5 ± 4.02).

This study was approved by the local ethics committee (Benha Faculty of Medicine Research Ethics Committee) and written informed consent, which were in compliance with the requirements of the Declaration of Helsinki, was obtained from all participants and the study was retrospectively registered on ClinicalTrials.gov (identifier, NCT05104294).

The clinical diagnosis of POAG was made after the demonstration of changes in the optic disk on clinical examination, e.g., optic nerve rim defect (localized thinning or notching), presence of visual field defects and open angle on gonioscopy. The pre-perimetric glaucoma eyes had characteristic glaucomatous optic disk changes, without presence of visual field defects.

The severity of POAG was determined according to visual field testing, patients with a global mean deviation (MD) less than − 6 dB were defined as "mild" POAG and patients with a global MD more than − 6 dB were defined as "moderate-severe" POAG.

Our inclusion criteria for both control and glaucoma groups were: age 30 years and older, spherical equivalent (SE) between − 2 and + 2 D, with no history of trauma or any previous ocular surgery, or systemic diseases. Normal healthy participants had normal ophthalmological examination, with non-glaucomatous optic disks, IOP less than 20 mm Hg and best corrected visual acuity (BCVA) of 6/9 or better.

Our exclusion criteria were as follows: SE more than + / − 2 diopters (D), history of any ocular surgeries, ocular trauma, optic nerve / macular or other retinal diseases, media opacity (cataract or corneal scarring), and unreliable visual field results (33% false positive, false negative or fixation losses).

### Ophthalmological examination

A complete clinical examination (slit-lamp examination, intraocular pressure (IOP) measurement by Goldmann applanation tonometry, refraction, BCVA (converted to LogMAR), and fundus examination) were underwent for all participants. Central corneal thickness (CCT) was measured by the LenStar LS900. OCTA was done using the RTVue XR OCT Avanti System AngioVue Version 201.2.0.93 (Optovue, Fremont, CA, USA). Peripapillary RNFL and GCC thickness were recorded from a 3.4 mm^2^ circle centered over the optic disk and the macular map scan, respectively. The superficial and deep capillary plexus vessel density (SCP-VD%, DCP-VD%) were measured by using of 6 mm × 6 mm macula OCTA scans {400 × 400 pixels (two repeats/B-scan), scan time 3 s, axial resolution 5 µm and transversal resolution 15 µm}. The peripapillary vessel density (RPC-VD%) was measured by using of 4.5 mm × 4.5 mm optic disk head OCTA scans. The new AngioVue software (Optovue, USA), incorporating the automated algorithms for mapping capillary density were applied to automatically measure the vessel density in the peripapillary area, superficial and deep capillary plexus. The vessel density (VD) was represented as a percentage by taking the ratio of the total vessel area to the total area of analyzed region. OCTA images with a signal strength index (SSI) more than 6 were included, any images with observable motion or defocus artifacts were excluded.

PhNR measurements were done “using RETI-port/scan 21(Roland Consult, Brandenburg, Germany),” PhNR implicit time and amplitude were recorded. The PhNR implicit time was calculated as the time interval from the onset of the stimulus to the peak of the negative wave. There are many techniques for PhNR amplitude measurements (Peak-to-trough: PT, Baseline-to-Trough: BT, Baseline-to-Fixed time: BF or b-wave/PhNR ratio) [16], but we preferred to use the BT technique (the difference between the pre-stimulus baseline and the trough of the negative wave following the b-wave) as it is the most reliable parameter of evaluation of RGC’s response and has direct correlation with it [17]. The PhNR examination was performed after Pupils dilation and after10 minutes of light adaptation and following the strict ISCEV (International Society for Clinical Electrophysiology of Vision) standards for the photopic electroretinogram.

PhNR was recorded using HK Loop electrodes which was installed into the lower fornix after applying topical anesthetic eye drops, ground gold skin electrodes attached on the central part of the forehead and the reference electrodes attached on the outer canthus skin of each eye. Ganzfeld settings were: red stimulus 0.4 cds/m^2^ (625 nm), on a blue background 25 cds/m^2^ (455 nm), with Inter-flash interval: 1 s.

### Statistical analyses

Software (SPSS, Version 26.0 for Windows, SPSS Inc., Chicago, IL) was used for the univariate, bivariate, and stratified analyses of the data. Qualitative variables were analyzed by constructing contingency tables with Pearson × 2 test. Analysis of variance (ANOVA) (with LSD as a post hoc test) and kruskal wallis test (KW) were used for multiple comparisons of quantitative variables for more than two groups. The Student t test and Mann–Whitney U test (as a post hoc test after KW analysis) were applied for the comparison of quantitative variables after establishing their normal distribution by means of the Shapiro–Wilk test and Levene test for equality of variance. Correlations among variables were studied by using the spearman coefficient for the association between variables. ROC (receiver operating characteristics) curve was performed to predict cutoff points for different variables. Differences were considered significant at *P* ≤ 0.05.

## Results

A total of 152 eyes were included 0.72 eyes with POAG (Glaucoma group), they were divided according to the mean deviation (MD) into two sub-groups 28 eyes with mild POAG {9 eyes were pre-perimetric} (group I), 44 eyes with moderate-severe POAG (group 2). The healthy control group included 80 eyes. There were no statistically significant differences between the glaucoma and control groups regarding age, gender, refraction or CCT (Table 1). There were significant differences in the C/D ratio, MD, IOP, RNFL and GCC thicknesses between the glaucoma and normal eyes (Table 1, 2, 3). There were significant reduction in the median Interquartile range (IQR) thickness of the GCC and RNFL in OAG patients versus normal (81.5 µm vs. 99 µm, *P* < 0.001; 90 µm vs. 108.5, *P* < 0.001, respectively). In the glaucoma patients the superior and inferior quadrants of RNFL were significantly thinner. More advanced POAG severity were associated with higher magnitudes of MD, increased cup-disk ratio and decreased both RNFL and GCC thicknesses (Table 2, 3).

**Table 1:** Demographic and clinical characteristics of normal and glaucomatous participants

		Glaucoma patients (*n* = 36) (72 eyes)	Control group (*n* = 40) (80 eyes)	Statistical test	*P* value
Age/years	Mean ± SD	40.5 ± 4.02	40.2 ± 4.39	St t = 0.31	0.76
Gender					
Male	N(%)	26 (72.2%)	24 (60.0%)	X^2^ = 1.26	0.26
Female		10 (27.8%)	16 (40.0%)		
IOP	Median (IQR)	22.5 (19.25 − 25.0)	13.0 (12.0 − 14.75)	MW = 10.25	< 0.001**
C/D ratio	Median (IQR)	0.7 (0.6 − 0.8)	0.4 (0.3 − 0.4)	MW = 10.46	< 0.001**
BCVA (log MAR)	Median (IQR)	0.55 (0.0 − 1.0)	0.0 (0.0 − 0.1)	MW = 6.82	< 0.001**
SE (D)	Median (IQR)	0.0 (− 2.0 − 1.0)	0.0 (− 1.0 − 1.0)	MW = 1.25	0.21
CCT (mm)	Median (IQR)	526.0 (514.0 − 542.25)	530.0 (520.0 − 539.75)	MW = 0.93	0.35

*BCVA* best corrected visual acuity; *CCT* central corneal thickness; *C/D* cup to disk ratio; *IQR* interquartile range; *IOP* intra ocular pressure; *SE* spherical equivalent; *St.’t’* Student’s t test; χ^2^ chi-squared test; *MW* Mann Whitney U test

*P value is significant; **P value is highly significant

**Table 2:** Comparison between the studied groups

		Group 1	Group 2	Control group	Statistical test (MW)	*P* value
MD	Median (IQR)	− 4.98 (− 5.32) − (− 4.43)	9.16 (− 12.14) − (− 8.04)$	− 0.78 (− 1.18) − (− 0.56)#^	124.41	< 0.001**
BCVA (Log MAR)	Median (IQR)	0.05 (0.0 − 0.38)	1.0 (0.6 − 1.0)$	0.0(0.0 − 1.0)#^	65.98	< 0.001**
SE	Median (IQR)	0.0 (− 1.0)–(1.0	− 1.0 (− 2.0) − (1.0)	0.0(− 1.0 − 1.0)	5.02	0.081
CCT (mm)	Median (IQR)	522.5 (513.0 − 536.0)	529.5 (515.0 − 543.0)	530.0(520.0 − 539.75)	2.44	0.30
IOP (mmHg)	Median (IQR)	18.5 (17.0 − 21.0)	24.0 (23.0 − 26.0)$	13.0(12.0 − 14.75)#^	116.62	< 0.001**
C/D ratio	Median (IQR)	0.6 (0.5 − 0.6)	0.8 (0.7 − 0.8)$	0.4(0.3 − 0.4)#^	122.93	< 0.001**

*BCVA* best corrected visual acuity; *CCT* central corneal thickness; *C/D* cup to disk ratio; *IOP* intra ocular pressure; *IQR* interquartile range; *MD* mean deviation; *SE* spherical equivalent; *MW* Mann Whitney U test

# = mild sig vs control; $ = mild sig Vs severe; ^ = severe sig versus control; **P value is highly significant

**Table 3:** Comparison between the studied groups regarding OCTA, OCT and PhNR Parameters

		Group 1	Group 2	Control group	Statistical test (MW)	*P* value
RPC –VD	Median (IQR)	52.9 (52.0 − 54.2)	46.15 (44.9 − 47.4)$	59.8 (58.63 − 61.29)#^	123.01	< 0.001**
SCP − VD	Median (IQR)	51.3 (49.9 − 53.6)	44.7 (43.1 − 48.1)$	54.8 (50.95 − 55.85)#^	78.22	< 0.001**
DCP − VD	Mean ± SD	50.74 ± 1.86	45.55 ± 2.43$	57.25 ± 4.19#^	F = 172.02	< 0.001**
Total RNFL	Median (IQR)	97.0 (92.0 − 102.0)	84.0 (70.0 − 90.0)$	108.5 (102.25 − 115.0)#^	107.63	< 0.001**
Inferior RNFL	Mean ± SD	103.79 ± 9.39	83.73 ± 14.10$	133.33 ± 14.74#^	F = 193.78	< 0.001**
Superior RNFL	Mean ± SD	103.93 ± 9.75	87.45 ± 13.28$	134.1 ± 14.23#^	F = 188.05	< 0.001**
Inferior GCC	Median (IQR)	90.0 (89.0 − 92.0)	76.0 (71.0 − 82.0)$	99.0 (95.25 − 101.75)#^	97.41	< 0.001**
Superior GCC	Median (IQR)	89.5 (89.0 − 90.0)	75.0 (73.0 − 83.0)$	99.0 (90.5 − 102.75)#^	86.56	< 0.001**
Total GCC	Median (IQR)	89.5 (87.0 − 90.0)	79.0 (77.0 − 82.0)$	99.0 (90.0 − 100.0)#^	91.55	< 0.001**
PhNR Amplitude	Median (IQR)	13.0 (12.0 − 15.0)	10.0 (9.1 − 11.68)$	19.9 (18.63 − 20.3)#^	121.09	< 0.001**
PhNR Implicit time	Mean ± SD	58.14 ± 1.83	62.0 ± 1.40$	51.82 ± 2.87#^	F = 280.89	< 0.001**

*DCP-VD* deep capillary plexus vessel density; *GCC* ganglion cell complex; *IQR* interquartile range; *MD* mean deviation; *PhNR* photopic negative response; *RNFL* retinal nerve fiber layer; *RPC-VD* peripapillary vessel density; *SCP-VD* superficial capillary plexus vessel density; F-test (ANOVA);*MW* Mann Whitney U test

# = mild sig vs control; $ = mild sig Vs severe; ^ = severe sig versus control; **P value is highly significant

**Fig. 1:**
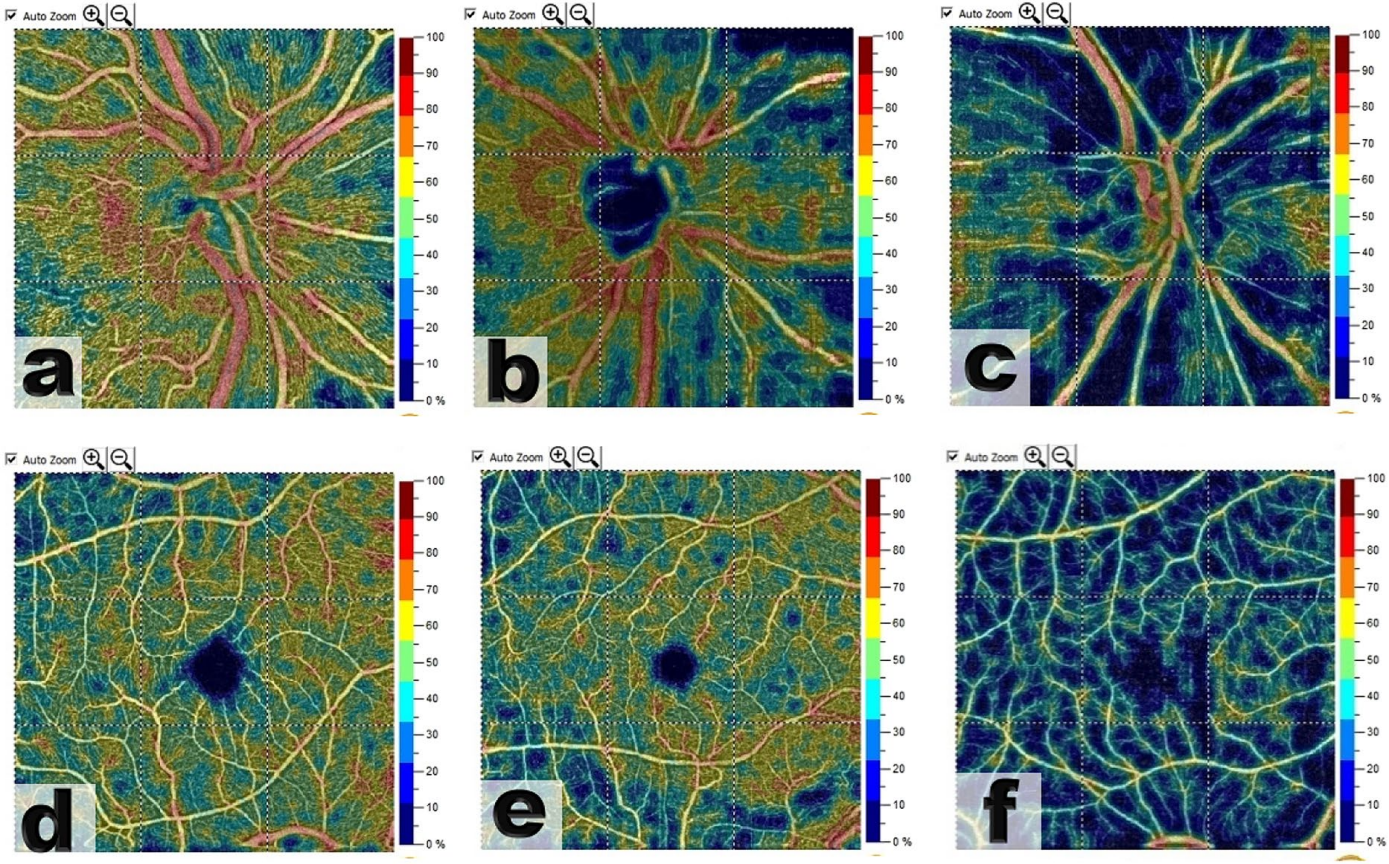
OCT-Angiography images {(4.5 × 4.5 mm OCTA optic disk head scans), (6 × 6 mm OCTA macula scans)}: with the corresponding color-coded vessel density (VD) mapping with quantitative data. In the POAG eyes the VD is reduced in comparison to healthy subjects with areas of non-perfusion. (**a**) RCP-VD in a healthy subject, (**b**) RCP-VD in a patient with mild POAG, (**c**) RCP-VD in a patient with severe POAG, (**d**) SCP-VD in a healthy subject, (**e**) SCP-VD in a patient with mild POAG, and (**f**) SCP-VD in a patient with severe POAG

**Fig. 2:**
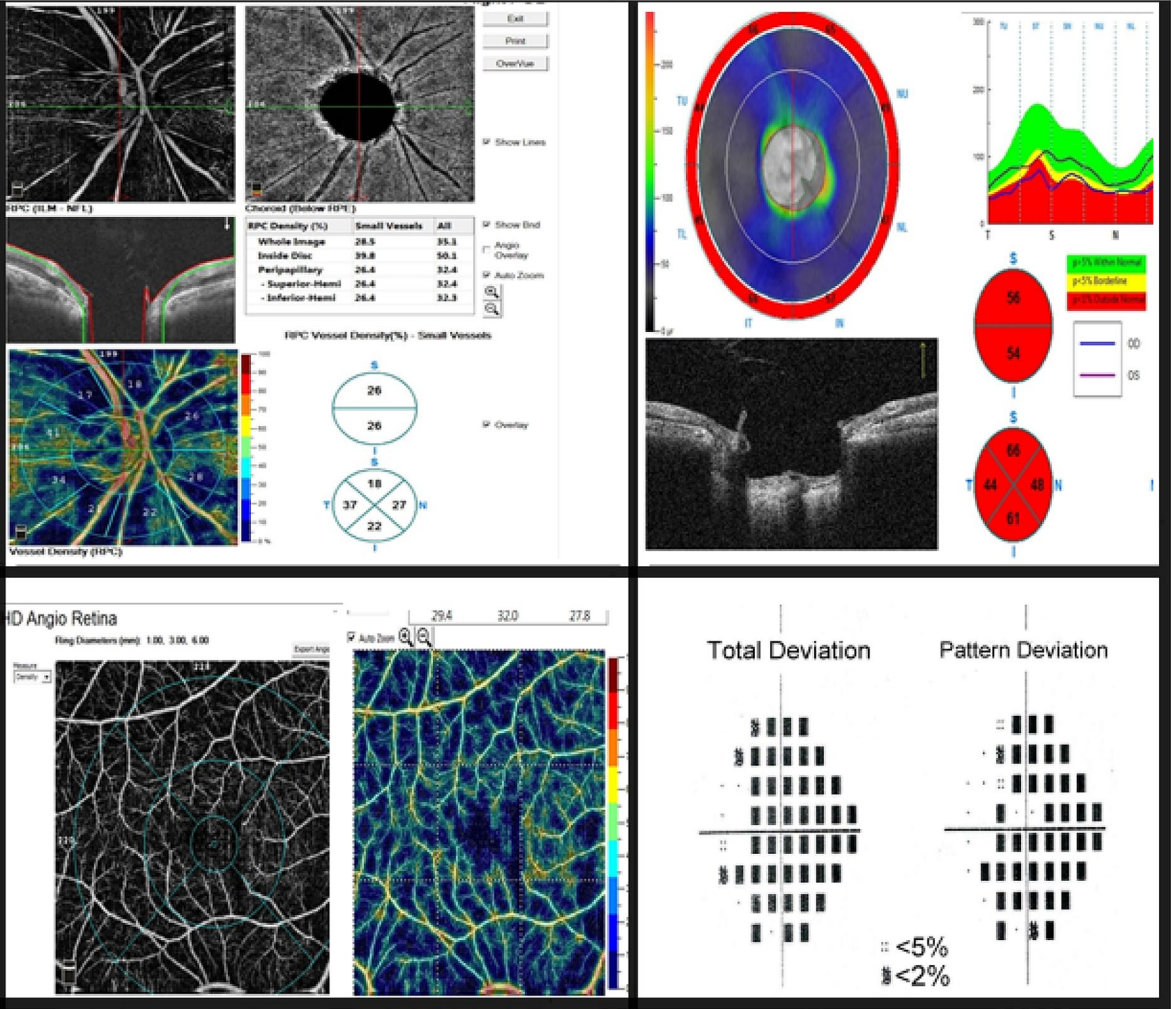
A case of moderate/severe POAG. Top and to the left (4.5 × 4.5 mm^2^ en-face optic disk head OCTA scans) and corresponding color-coded vessel density (VD) mapping. OCT RNFL deviation map and RNFL thickness by quadrant (top and to the right). Bottom and to the left (6 × 6 mm en-face macula OCTA and corresponding color-coded VD mapping scans). Probability total and pattern deviation maps (Bottom and to the right). The VD is reduced in eyes with POAG with areas of non-perfusion

**Fig. 3:**
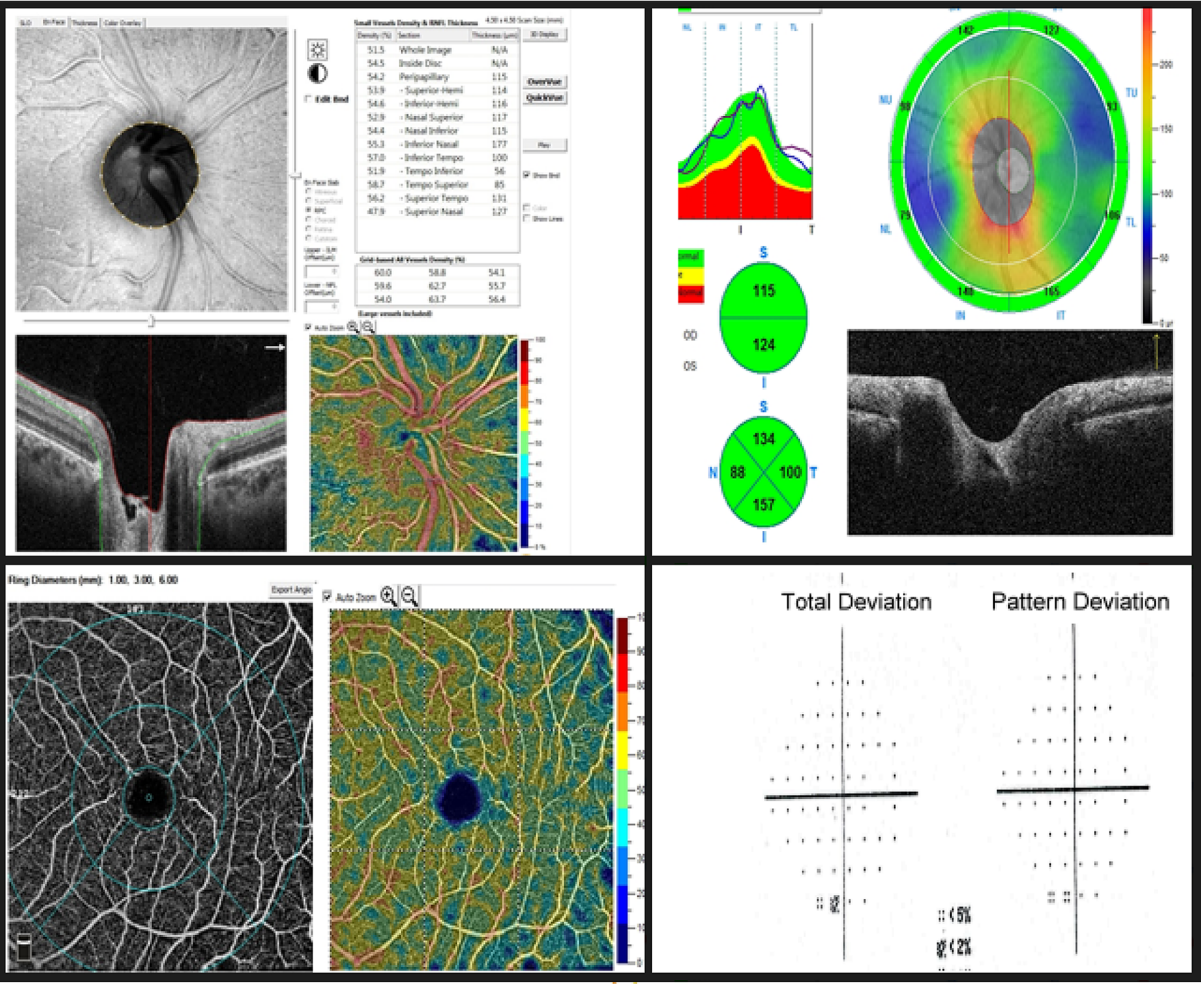
A healthy control subject. Top and to the left (4.5 × 4.5 mm.^2^ en-face optic disk head OCTA scans) and corresponding color-coded vessel density (VD) mapping with areas of higher vessel density in warmer colors. OCT RNFL deviation map and RNFL thickness by quadrant (top and to the right). Bottom and to the left (6 × 6 mm en-face macula OCTA and corresponding color-coded VD mapping scans). Probability total and pattern deviation maps (Bottom and to the right)

RPC-VD%, SCP-VD % and DCP-VD% were significantly reduced in OAG patients versus normal (48.15% vs. 59.8%, *P* < 0.001, 47.85% vs. 54.8%, *P* < 0.001; 47.57% vs. 57.25%, *P* < 0.001; respectively), with a trend of worsening with increased POAG severity, (Figs. 1, 2, 3) (Table 3).

**Fig. 4:**
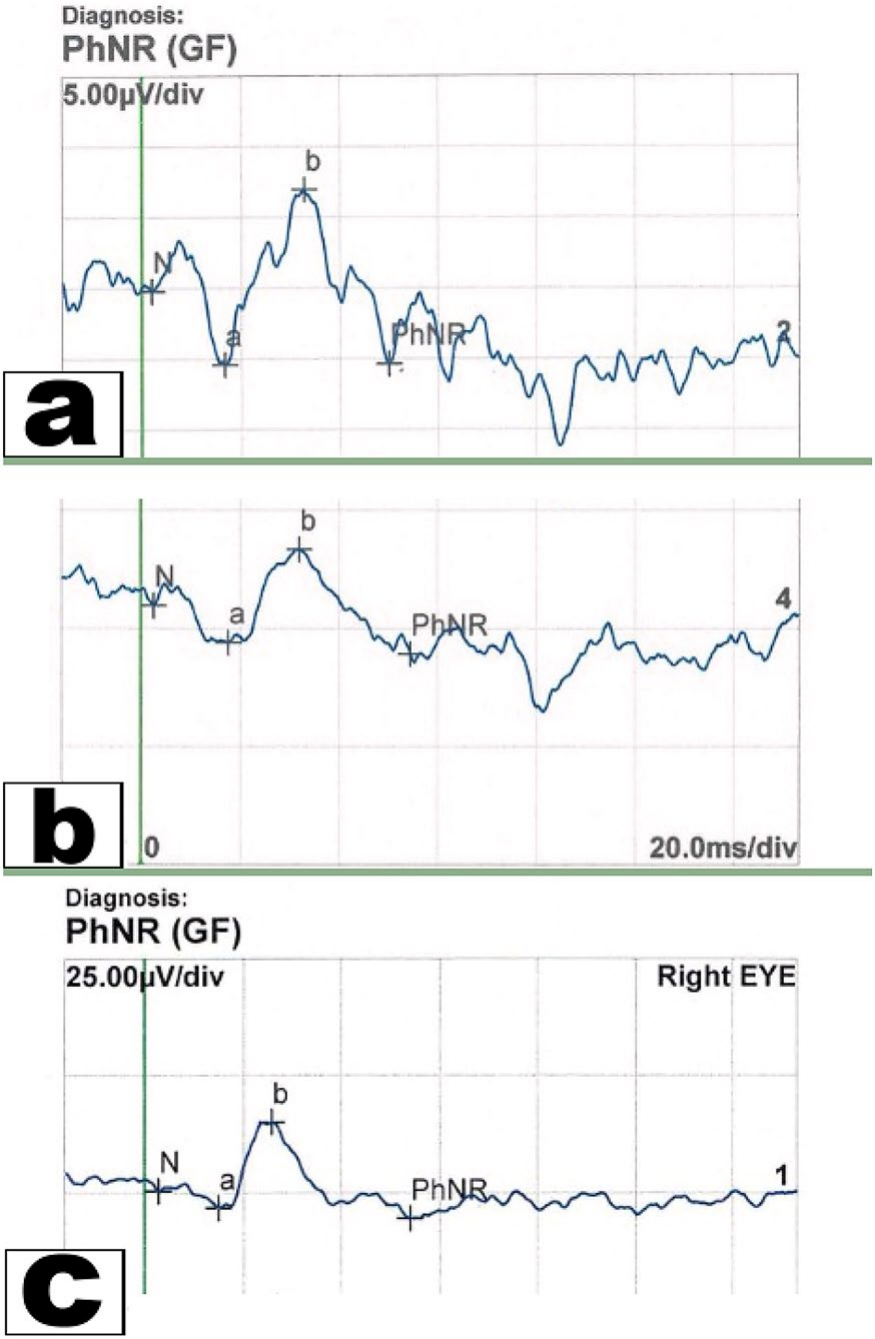
PhNR images. The PhNR amplitude is reduced and the PhNR implicit time is increased in eyes with POAG in comparison to controls (**a**) PhNR in a healthy subject, (**b**) PhNR in a patient with mild POAG, (**c**) PhNR in a patient with severe POAG

There were statistically significant differences of PhNR implicit time and amplitude between OAG patients versus normal (60.5 vs. 51.82, *P* < 0.001; 11.7 vs. 19.9, *P* < 0.001, respectively), increased POAG severity was associated with more reduction in PhNR amplitude and prolonged latency, (Fig. 4) (Table 3). The strength of association of each vascular parameter with structural (RNFL & GCC thicknesses) and functional (MD & PhNR) measures were evaluated in mild and moderate /severe POAG groups. In group (1) the RCP-VD demonstrated significant positive correlations with RNFL thickness, PhNR amplitude and MD (*P* < 0.001; rho = 0.610, *P* = 0.003; rho = 0.539, *P* = 0.003; rho = 0.535, respectively), there were also significant negative correlation with PhNR implicit time (*P* < 0.001; rho =  − 0.869); however, the correlation remained insignificant with GCC thickness (*P* = 0.356; rho =  − 0.181). In group (1) the SCP-VD demonstrated significant correlations with RNFL thickness, PhNR implicit time (*P* = 0.002; rho = 0.567, *P* < 0.001; rho =  − 0.777, respectively); however, the correlation remained insignificant with MD, PhNR amplitude and GCC thickness (*P* = 0.053; rho =  − 0.370; *P* = 0.220; rho =  − 0.240; *P* = 0.481; rho =  − 0.139, respectively). In group (2) both RCP-VD and SCP-VD demonstrated significant associations with both structural and functional parameters, Table 4.

**Table 4:** Correlations between vascular parameters and different structural and functional parameters in POAG patients

Variables	RPC-VD				SCP-VD			
	Group 1		Group 2		Group 1		Group 2	
	*Rho*	*P*	*Rho*	*P*	*Rho*	*P*	*Rho*	*P*
MD	0.535	0.003**	0.513	< 0.001**	0.370	0.053	0.472	0.001**
PhNR (amplitude)	0.539	0.003**	0.836	< 0.001**	0.240	0.220	0.669	< 0.001**
PhNR (implicit time)	− 0.869	< 0.001**	− 0.813	< 0.001**	− 0.777	< 0.001**	− 0.726	< 0.001**
RNFL (Total)	0.610	0.001**	0.740	< 0.001**	0.567	0.002**	0.667	< 0.001**
GCC (Total)	0.181	0.356	0.611	< 0.001**	0.139	0.481	0.734	< 0.001**

*GCC* ganglion cell complex; *MD* mean deviation; *RNFL* retinal nerve fiber layer; *phNR* photopic negative response; *RPC-VD* peripapillary vessel density; *rho* Correlation Coefficient Spearman's; *SCP-VD* superficial capillary plexus vessel density

*P value is significant;**P value is highly significant

Although the PhNR amplitude was not significantly correlated with the MD in mild POAG, there were significant correlations between it and RCP-VD, RNFL and GCC thicknesses (*p* = 0.003, 0.020, 0.026, respectively). In patients with moderate /severe POAG, both PhNR amplitude and implicit time demonstrated significant associations with the vascular and structural parameters, which indicates that the function of the inner retina reduced proportionately with the neural loss, Table 5.

**Table 5:** Correlations between PhNR parameters and different structural and vascular parameters in POAG patients

Variables	PhNR (amplitude)				PhNR (implicit time)			
	Group 1		Group 2		Group 1		Group 2	
	*Rho*	*P*	*Rho*	*P*	*rho*	*P*	*Rho*	*P*
MD	0.19	0.922	0.442	0.003**	− 0.380	0.046*	− 0.562	0.001**
RNFL (Total)	0.439	0.020*	0.776	< 0.001**	− 0.601	0.001**	− 0.810	< 0.001**
GCC (Total)	0.419	0.026*	0.357	0.017*	− 0.73	0.713	− 0.346	0.021*
RCP − VD	0.539	0.003**	0.836	< 0.001**	− 0.869	< 0.001**	− 0.813	< 0.001**
SCP − VD	0.240	0.220	0.669	< 0.001**	− 0.777	< 0.001**	− 0.726	< 0.001**

*GCC*—ganglion cell complex; *MD*—mean deviation; *PhNR*—photopic negative response; *rho* Correlation Coefficient Spearman's; *RNFL*—retinal nerve fiber layer; *RPC-VD*—peripapillary vessel density; *SCP-VD*—superficial capillary plexus vessel density

^*^P value is significant; **P value is highly significant

Table 6 shows the diagnostic accuracy of each vascular, structural and functional parameter as measured by AUC. The RCP-VD had the ROC curve with the AUC value (1.0, *P* < 0.001), the sensitivity and specificity for a cutoff point of 57.55 were 100% and 97.5%, respectively, with a diagnostic accuracy 98.7. The PhNR amplitude had the ROC curve with the AUC value (1.0, *P* < 0.001), the sensitivity and specificity for a cutoff point of 15.45 were 100% and 97.5%, respectively, with a diagnostic accuracy 98.7, followed by the PhNR implicit time with (AUC = 0.995) with a diagnostic accuracy 98.7.

**Table 6:** ROC curve of RCP- VD, SCP-VD, DCP-VD, PhNR amplitude/implicit time, RNFL&GCC thicknesses between POAG and healthy eyes

Case and control	RCP − VD	SCP − VD	DCP − VD	PhNR Amplitude	PhNR Implicit time	RNFL total	GCC total
AUC	1.0	0.868	0.945	1.0	0.995	0.959	0.915
95% CI	1.0 − 1.0	0.814 − 0.922	0.913 − 0.977	1.0 − 1.0	0.988 − 1.0	0.934 − 0.985	0.874 − 0.956
Cut − off point	57.55	53.25	53.4	15.45	55.31	105.5	93.0
Sensitivity	100	86.1	100	100	100	100.0	100
Specificity	97.5	65.0	80.0	97.5	97.5	65.0	70.0
PPV	97.3	68.9	81.8	97.3	97.3	72.0	75.0
NPV	100	83.9	100	100	100	100	100
Accuracy	98.7	75.0	69.7	98.7	98.7	81.6	84.2

*AUC*—Area under the curve; *DCP-VD*—deep capillary plexus vessel density; *GCC*—ganglion cell complex; *MD*—mean deviation; *NPV*—Negative predictive values; *PhNR*—photopic negative response; *PPV*—positive predictive values; *rho* Correlation Coefficient Spearman's; *RNFL*—retinal nerve fiber layer; *RPC-VD*—peripapillary vessel density; *SCP-VD*—superficial capillary plexus vessel density

^*^P value is significant; **P value is highly significant

Diagnostic accuracy was (75.0, 81.6 and 84.2) for SCP-VD, RNFL and GCC thickness, respectively (*P* < 0.001).

**Fig. 5:**
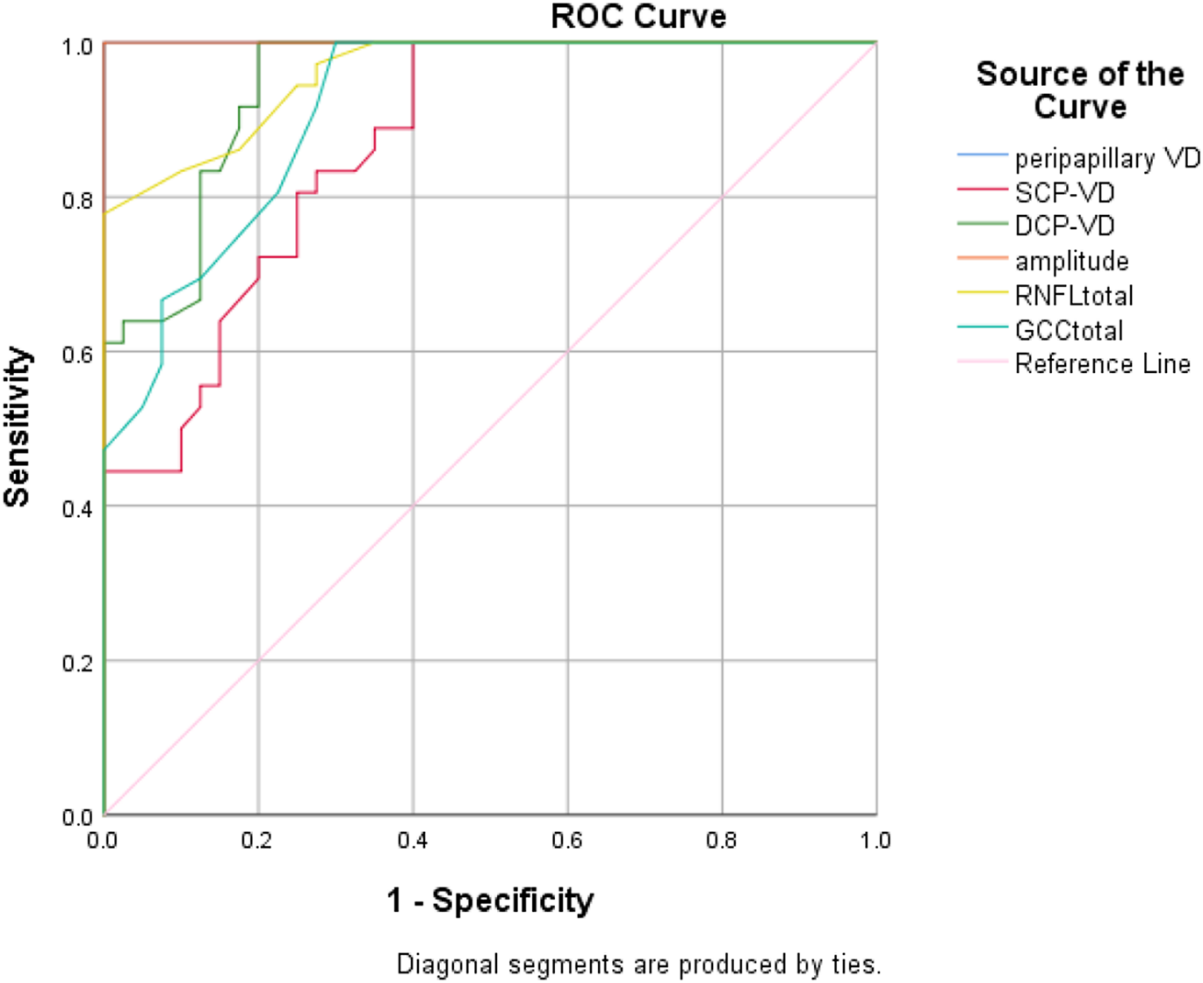
ROC curve for OCTA parameters, PhNR, RNFL&GCC thicknesses between POAG and healthy eyes

The ROC curve showed largest AUC in RCP-VD and PhNR amplitude with higher sensitivity and specificity (100% and 98.7%, respectively) (Fig. 5).

## Discussion

Imaging plays a very important role in early detection and management of glaucoma [1, 4]. It is important to develop methods for evaluating POAG because there are few ways to objectively evaluate optic nerve function. OCT-angiography (OCTA) and PhNR are minimally-invasive techniques that can provide evaluation and useful measurements of the retina and optic nerve head (ONH). OCTA can monitor the vessel perfusion and blood flow within the retina and ONH and evaluate the health of the RGCs and their axons that can be damaged in OAG [2, 8, 11]. PhNR does not need patient cooperation, refractive correction or clear ocular media as in visual field testing & has direct correlation with RGCs function [16, 17].

In the current study we evaluated the vascular, functional and structural changes in the peripapillary and macular area in patients with POAG.

There were significant differences in RNFL, GCC thicknesses and visual field MD between the healthy and POAG eyes. Glaucomatous eyes had significant reduction in the inner plexiform layer of macular ganglion cells (GCIPL) and peripapillary RNFL microcirculation. These results are in agreement with previous studies [2, 9, 18].

Regarding PhNR parameters, eyes with POAG had marked reduced amplitude and increased implicit time (IT) in comparison to normal eyes, these results are in accordance with a study by Banerjee et al. [25] who compared 25 glaucoma patients with 50 healthy controls, and they also reported that PhNR amplitude was significantly reduced in glaucoma eyes, while (IT) was significantly increased. Preiser et al. [26] in their study also reported that PhNR can be affected in eyes with pre-perimetric glaucoma. Sustar et al. [27] also observed a statistically significant differences in PhNR amplitude between OAG and normal eyes (using the same BT measurements of PhNR amplitude), and they assumed that PhNR can distinguish between OAG and healthy eyes with 92.9% specificity and 92% sensitivity.

We also assessed the strength of association of each vascular perfusion parameters with structural (RNFL & GCC thicknesses) and functional (MD & PhNR) measures in different POAG groups. In patients with mild POAG, both RCP-VD & SCP-VD demonstrated a significant correlation with RNFL thickness, the amplitude of PhNR and IT; however, the correlation remained insignificant with the thickness of the GCC. The PhNR amplitude was not significantly correlated with MD in mild POAG; however, there was a significant correlation between it and RCP-VD, RNFL and GCC thicknesses (*p* = 0.003, 0.020, 0.026, respectively). The linear relationship between the PhNR and the vascular parameters indicates that inner retinal function declines proportionately with changes in microvasculature in eyes with OAG. We assumed that in the early OAG, the decrease in peripapillary and macular vessel density is significantly associated with the severity of functional loss, regardless of structural loss. Previous studies on RNFL-GC-IPL perfusion also reported that OCTA -VD in the macular area was better associated with functional visual field parameters than structural GCC thickness [9, 28].

In patients with moderate to severe POAG, while all correlations were significant, the associations of vascular parameters with functional measures were qualitatively higher than structural correlations. These observations were like that in Richard et al.[9], who also reported that OCTA-VD in the macular area was better correlated with functional parameters, with no significant correlations with structural measures. However, other studies have demonstrated that structural correlations are stronger than functional ones [18, 22–24]. Richard and his colleague[18] in their study reported that the associations of perfusion OCTA parameters were significantly higher with RNFL structural measures than visual field functional associations. These differences may be due to usage of different segmentation techniques, scan sizes, and vascular parameters; however, longitudinal studies of disease progression are still needed to explicate these controversies.

In POAG patients, we noticed that macular VD was decreased compared to control subjects in the superficial and the deep capillary plexus as well, indicating changes in the retinal microvasculature at different levels, but this observation deserves further study.

In the current study, we also evaluated the diagnostic accuracy of different OCTA and PhNR parameters for the detection of glaucoma. The RCP-VD and PhNR amplitude demonstrated a higher diagnostic ability (98.7) with the highest AUC and higher sensitivity and specificity (100% and 98.7%, respectively), followed by the PhNR implicit time (AUC = 0.995) with a diagnostic accuracy of 98.7. The SCP-VD, RNFL and GCC thicknesses had a diagnostic accuracy of (75.0, 81.6 and 84.2), respectively (*P* < 0.001). These results reflect a better diagnostic performance of the peripapillary vascular parameters than the macular vascular parameters for the diagnosis of glaucoma which are consistent with previous studies [2–29].

In the current study there was equivalent diagnostic ability between peripapillary OCTA parameters and PhNR, so both can detect early microvasculature & functional changes in GCl and their axons even before structural measurements, and both may be a promising supplement for current glaucoma diagnostic tools.

We think that there is some novelty in this study from the perspective of correlation between OCTA and the PhNR. The limitations of the current study were the relatively small sample size, we did not evaluate the effect of glaucoma medications on OCTA or PhNR parameters, as both glaucoma groups were on similar medications; however, further longitudinal studies involving larger numbers of subjects are needed to assess the association between various glaucoma treatment and these parameters.

In conclusion: Both OCTA and PhNR are perfect biomarkers for glaucomatous damage, the PhNR may represent a useful additional tool in glaucoma diagnosis, decreased PhNR parameters indicate inner retinal dysfunction that may precede the neural loss in early stages of glaucoma. The vascular OCTA parameters and PhNR (an objective measure of ganglion cell function) can supplement the structural OCT measurement if it has limited diagnostic value, as in high myopia, for the diagnosis and monitoring of POAG.

## Data Availability

The data of patients used to support the results of this study are limited by the Research Ethics Committee of the Faculty of medicine, Benha University. Data are available to researchers who meet the criteria for accessing confidential data at the request of Dr. Ahmed Abdelshafy Tabl, lecturer of Ophthalmology, Benha University, Egypt. E-mail: ahmad4Lg@gmail.com.
